# Role of the Crosstalk between Autophagy and Apoptosis in Cancer

**DOI:** 10.1155/2013/102735

**Published:** 2013-06-05

**Authors:** Minfei Su, Yang Mei, Sangita Sinha

**Affiliations:** Department of Chemistry and Biochemistry, North Dakota State University, P.O. Box 6050, Dept. 2710, Fargo, ND 58102-6050, USA

## Abstract

Autophagy and apoptosis are catabolic pathways essential for organismal homeostasis. Autophagy is normally a cell-survival pathway involving the degradation and recycling of obsolete, damaged, or harmful macromolecular assemblies; however, excess autophagy has been implicated in type II cell death. Apoptosis is the canonical programmed cell death pathway. Autophagy and apoptosis have now been shown to be interconnected by several molecular nodes of crosstalk, enabling the coordinate regulation of degradation by these pathways. Normally, autophagy and apoptosis are both tumor suppressor pathways. Autophagy fulfils this role as it facilitates the degradation of oncogenic molecules, preventing development of cancers, while apoptosis prevents the survival of cancer cells. Consequently, defective or inadequate levels of either autophagy or apoptosis can lead to cancer. However, autophagy appears to have a dual role in cancer, as it has now been shown that autophagy also facilitates the survival of tumor cells in stress conditions such as hypoxic or low-nutrition environments. Here we review the multiple molecular mechanisms of coordination of autophagy and apoptosis and the role of the proteins involved in this crosstalk in cancer. A comprehensive understanding of the interconnectivity of autophagy and apoptosis is essential for the development of effective cancer therapeutics.

## 1. Introduction to Autophagy

Autophagy is a cell-survival pathway conserved in all eukaryotes. It involves the selective degradation of cellular components, including long-lived proteins, protein aggregates, damaged cytoplasmic organelles, and intracellular pathogens, resulting in the recycling of nutrients and the generation of energy [1]. Basal levels of autophagy are required for cellular homeostasis. Autophagy is upregulated under stress conditions, including extracellular stress such as nutrition deprivation, hypoxia, and infection and intracellular stress such as that caused by accumulation of damaged proteins and organelles and high bioenergetic demands. It allows lower eukaryotes to survive starvation, while in mammals, it is thought to be involved in many physiological and pathophysiological processes, including antiaging mechanisms, differentiation and development, immunity, and elimination of microorganisms [2–9].

**Figure 1:**
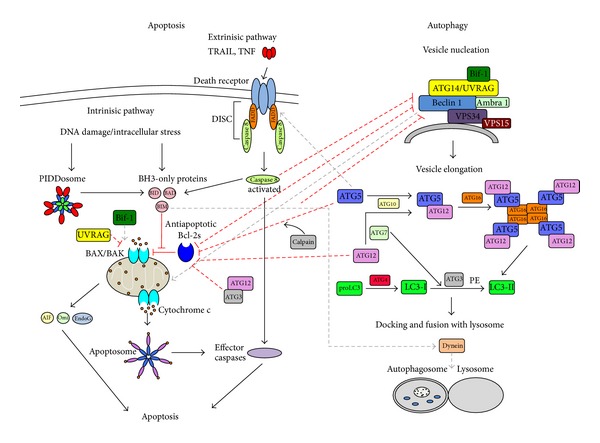
Crosstalk between autophagy and apoptosis. Various proteins involved at the different points of crosstalk are shown and labeled. Lines denote interactions or processes, with solid lines corresponding to intrapathway processes and dashed lines corresponding to inter-pathway connections. Red lines denote inhibitory interactions, while lines with arrows indicate facilitating interactions.

Autophagy is a highly regulated process (Figure 1) executed by autophagy-related effectors, many of which are called ATG proteins [1, 6, 10]. The first committed step of autophagy is vesicle nucleation in which macromolecular assemblies selected for degradation are surrounded by isolation membranes called phagophores. The vesicle nucleation process is executed by a protein complex whose core comprises the class III phosphatidylinositol-3-kinase (PI3Kc3 or VPS34) which catalyzes phosphorylation of phosphatidylinositol to phosphatidylinositol 3-phosphate; the PI3Kc3 regulatory subunit (p150 or VPS15), a myristylated serine/threonine kinase that phosphorylates PI3Kc3 and recruits it to the membrane; and the BCL-2 interacting protein (Beclin 1 or ATG6), which appears to be a protein interaction hub. More recently, Ambra 1, identified as a positive regulator of autophagy that interacts with Beclin 1, was shown to be part of the core complex [11]. Further, the core complex variably associates with various other proteins such as ATG14, UV radiation resistance-associated gene (UVRAG), vacuole membrane protein 1 (Vmp1), endophilin B1 (Bif-1), and Beclin 1 associated RUN domain containing protein (Rubicon), forming complexes that have distinct functions in membrane trafficking processes [12, 13].

Membranes comprising the nascent phagophore are enlarged and then fused at their edges to form multi-layered vesicles called autophagosomes. Two ubiquitin-like conjugation systems are involved in this process [14–17]. In one system, ATG12, a protein with a ubiquitin-like fold, is covalently conjugated to ATG5 by the activity of an E1-like enzyme, ATG7, and an E2-like enzyme, ATG10. The ATG12-ATG5 conjugate then forms a larger multimeric complex with ATG16. In the other system, another ubiquitin-like protein, ATG8/LC3, is conjugated to phosphatidylethanolamine, by the sequential action of a protease, ATG4, the E1-like enzyme, ATG7, and an E2-like enzyme, ATG3. The outer membrane of autophagosomes eventually fuses with lysosomes to create autophagolysosomes. This stage involves specific ATG8 paralogs; multiple WD repeat domain-containing, phosphoinositide-interacting ATG18 paralogs; ATG2A, which binds to ATG18; the transmembrane protein, ATG9; SNX18 which causes autophagosome tubulation; as well as small G-proteins that direct this process. Inside the autophagolysosome, the contents of the autophagosome are degraded by lysosomal enzymes, after which the degradation products are recycled by the cell.

## 2. Introduction to Apoptosis 

Apoptosis is the best-understood mechanism of programmed cell death. It is recognized by distinct morphological characteristics of cells, such as cellular shrinkage with nuclear chromatin condensation and nuclear fragmentation. It functions as a homeostasis mechanism to maintain cell populations, as well as a defense mechanism in the presence of toxic agents. Apoptosis can be triggered by diverse cellular signals. These include intracellular signals produced in response to cellular stresses, such as increased intracellular Ca^2+^ concentration, oxidative damage caused by reactive oxygen species (ROS) [18], and hypoxia [19]. Extrinsic inducers of apoptosis include bacterial pathogens [20], toxins [20], nitric oxide [21], growth factors [22], and hormones [23]. 

Depending on the apoptosis-inducing signal, two different apoptosis pathways have been identified (Figure 1): the intrinsic pathway characterized by mitochondrial outer membrane permeabilization (MMP) and the release of mitochondrial cytochrome c; and the extrinsic pathway, which is initiated by death-receptor stimulation. There is an overlap between these pathways as the extrinsic pathway usually also activates the intrinsic pathway (Figure 1), and both pathways result in the recruitment and activation of cysteine-aspartic acid proteases (caspases) [24, 25]. Intracellular apoptotic signals trigger the intrinsic pathway, starting with the activation of different BCL-2 homology 3 (BH3) domain-only proteins. Activated BH3-only proteins bind to antiapoptotic BCL-2 proteins, preventing them from binding to and inhibiting the multi-BH domain proapoptotic proteins, BAX and BAK. This allows homodimerization of BAX and/or BAK in the outer mitochondrial membrane, forming channels that increase MMP to permit the release of cytochrome c as well as other apoptosis effectors. Cytochrome c then associates with apoptotic protease-activating factor 1 (APAF-1) and caspase-9 to form a complex called the apoptosome. The apoptosome activates effector caspases leading to cell death [26, 27]. Intracellular stress such as DNA damage results in the transcriptional upregulation of proapoptotic proteins like p53-induced protein with a death domain (PIDD). PIDD recruits receptor-interacting protein (RIP)-associated ICH-1/CED-3 homologous protein with a death domain (RAIDD) and caspase 2 to form a 700 kDa complex called PIDDosome [17]. Caspase 2 activated inside the PIDDosome induces apoptosis via cleavage of BH3-only proteins like BID, and subsequent MMP. The extrinsic pathway starts with the stimulation of specific death receptors upon binding of their ligands, like tumor necrosis factor-related apoptosis-inducing ligand (TRAIL) and tumor necrosis factor (TNF). The binding of ligands causes trimerization of these death receptors, resulting in clustering of their death domains and recruitment of Fas-associated death domain (FADD) and caspase 8, to form the death-inducing signaling complex (DISC) [28]. Caspase 8 is activated inside the DISC and can then promote cell death, either by activating effector caspases or by cleaving the BH3-only protein BID to initiate mitochondria-dependent apoptosis [26, 27].

## 3. The Molecular Crosstalk between Autophagy and Apoptosis May Be Important in Cancer

Autophagy and apoptosis are both cellular degradation pathways essential for organismal homeostasis. Therefore, it is not surprising that both autophagy and apoptosis have been implicated in protecting organisms against a variety of diseases, especially cancer [29–31].

### 3.1. Apoptosis Is a Tumor Suppressor Pathway

Apoptosis is estimated to eliminate approximately 60 billion cells per day for organismal homeostasis. Deregulation of apoptosis leads to accumulation of “unwanted” cells and contributes to cancer development. Numerous disruptions in apoptosis signaling pathways, including both the extrinsic and intrinsic pathways, have been observed in cancer cells. The extrinsic apoptotic pathway is often disrupted due to inhibition of signaling from the death receptors. Such signaling inhibition, which has been implicated in a variety of cancers, includes mutations in death receptors and changes in death receptor expression or localization, such as the downregulation of surface expression of death receptor, epigenetic changes, and overexpression of decoy receptors [32, 33]. Genetic alterations are the most common cause of defects in the intrinsic apoptosis pathway that result in cancer [32]. For example, chromosomal translocation of the antiapoptotic *bcl-2* oncogene is found to be associated with most human follicular lymphoma. Inactivation of the proapoptotic *bax* gene is implicated in some solid tumors and hematological malignancies. Mutations of proapoptotic BH3-only protein genes have also been found to contribute to cancer development. Besides the BCL-2 protein family, the tumor repressor p53 is frequently mutated in cancer cells [32]. Therefore, a long-standing goal of anticancer therapeutics is to upregulate apoptosis within cancer cells to cause the death of these cells. Indeed, current anticancer treatments, including many chemotherapeutic agents as well as ionizing radiation therapy, actually activate apoptosis to utilize the apoptotic machinery to kill cancer cells [33–35].

### 3.2. Autophagy: A Double-Edged Sword in Cancer

As autophagy removes damaged proteins and organelles, it limits their cumulative deleterious effects inside cells. Therefore, it is not surprising that autophagy defects are found in many human tumors [36–38]. Further, excessive autophagy has been implicated in autophagic cell death, also called type II cell death, which is characterized by morphologic changes such as the accumulation of autophagosomes inside the cell. The exact molecular mechanism of type II cell death is unknown, although many autophagy proteins are implicated in this process [39]. 

In contrast to these tumor-suppressor roles for autophagy, stress-activated autophagy may promote survival of tumor cells, especially when apoptosis is defective. Hypoxia has previously been reported to select cells with defective apoptosis in solid tumors [40]. More recently, it was shown that autophagy localizes to unvascularized, metabolic-stressed regions of tumors [41]. In hypoxia and nutrient-limited solid tumor centers, starvation-activated autophagy may promote cell survival by breaking down cellular building blocks to provide the missing nutrients. Thus, autophagy appears to play a dual role in cancer. For instance, tumorigenesis is suppressed by Beclin 1 expression in human MCF7 breast cancer cells [42]. While *beclin 1* ^−/−^ mice die early in embryogenesis, mammary tissue from *beclin 1* ^+/-^ mice shows hyperproliferative, preneoplastic changes [43], and aging *beclin 1* ^+/-^ mice have an increased incidence of lymphoma and carcinomas of the lung and liver [43, 44]. However, despite such clear evidence that autophagy prevents cancer development, it has also been shown that amongst immortalized, apoptosis-defective mouse mammary epithelial cells, *beclin 1* ^+/+^ cells are more resistant to cell death upon nutrient and oxygen deprivation and survive longer compared to *beclin 1* ^+/-^ cells, suggesting that autophagy may be activated to promote cell survival in apoptosis-defective cells [37, 45]. Further, autophagy may facilitate survival of a small number of tumor cells that manage to tolerate damage and stress induced by cancer treatment, which may then reemerge at a later time, constituting a fundamental barrier to successful cancer treatment [46, 47].

### 3.3. The Crosstalk between Autophagy and Apoptosis

Since both autophagy and apoptosis play multiple, essential roles in cellular homeostasis, it is perhaps not surprising that there is extensive crosstalk between them that enables the coregulation of these pathways (Figure 1). Nodes of crosstalk include the Beclin 1-BCL-2 interaction [48]; caspase-mediated Beclin 1 cleavage [49–51]; UVRAG-BAX interaction [52]; ATG12-ATG3 conjugation [53]; ATG12-Mcl-1 interaction [54]; ATG5-FADD interaction [55]; Calcium-dependent, nonlysosomal, cysteine protease- (Calpain-) mediated ATG5 cleavage [56]; tumor protein 53- (p53-) mediated cross-regulation [57, 58]. As inhibition of both autophagy and apoptosis has been shown to cause cancer, it is likely that proteins involved in the crosstalk between these pathways may have particularly important roles in this disease. In the subsequent sections, we describe the complex molecular crosstalk between autophagy and apoptosis and the role of these proteins in cancer. Such a holistic view of cellular processes, combined with detailed molecular information about the mechanisms of crosstalk, is crucial for the successful future development of anticancer therapeutics.

## 4. Beclin 1

Beclin 1 is an essential autophagy effector that has important roles in the cross-talk with the apoptosis pathway. Human Beclin 1 is a 450-amino acid protein that contains three domains of known structure: a BH3 domain, (residues 108-127) [9, 59–62], a coiled-coil domain (residues 175-265) [63], and a C-terminal evolutionarily conserved domain (residues 248-450) [64].

### 4.1. The Beclin 1-BCL-2 Interaction

Beclin 1 was first discovered as a protein that interacts with the antiapoptotic BCL-2 proteins [48] and only later shown to associate with PI3Kc3 and p150 to form the vesicle nucleation complex essential for autophagy [65, 66]. Thus, the Beclin 1-BCL-2 interaction was the first established molecular connection between autophagy and apoptosis. Both Beclin 1 and BCL-2 have established roles in the development of cancer. Beclin 1 was found to be monoallelically deleted in 40% of sporadic human breast cancers [67], establishing the first functional link between autophagy and cancer. Overexpression of antiapoptotic BCL-2 proteins has long been shown to correlate with resistance to chemotherapy and radiotherapy in various cancers [68–70]. Indeed, cancers arising due to defects in BCL-2 were the first cancers shown to arise due to defective cell death, rather than due to defective cell duplication [71–75]. 

The BCL-2 family proteins are recognized by the presence of poorly-conserved BH domains. This family includes several, BH3-only, proapoptotic proteins such as BIM and BAD; at least three multi-domain (BH1, BH3, BH2), proapoptotic proteins, BAX, BAK and BOK; and at least six multi-BH domain (BH4, BH1, BH3, BH2) antiapoptotic proteins, BCL-2, BCL-X_L_, MCL-1, BCL-w, A1 and BCL-B. The antiapoptotic BCL-2 homologs bind to different BH3Ds with widely varying affinities, which dictates differential specificity of interaction. Beclin 1 has been shown to bind via its BH3 domain to various BCL-2 homologs [59–62]. This interaction appears to help maintain autophagy at levels essential for normal cellular homeostasis, while mutations in Beclin 1 that block the interaction with BCL-2 prevent BCL-2 from inhibiting autophagy [76, 77]. Thus, the Beclin 1- BCL-2 interaction provides an important node of crosstalk between apoptosis and autophagy [60].

Defects in either BCL-2 or Beclin 1 affect both autophagy and apoptosis. For instance, increased Beclin 1 expression may release BAK/BAX from BCL-2 to promote apoptosis, while decreased BCL-2 expression may result in excessive Beclin 1-dependent autophagy. Indeed, Beclin 1 overexpression has been shown to increase anticancer drug-induced apoptosis in cervical cancer cells, thus sensitizing cancer cells to chemotherapeutic drugs [78]. Inhibition of BCL-2 expression has also been reported to elevate Beclin 1 levels and result in the death of breast cancer cells [79].

As BCL-2 can regulate Beclin 1-induced autophagy, as well as, mitochondria-dependent apoptosis by direct binding to Beclin 1 and BAX/BAK, any drug that inhibits BCL-2 would be able to upregulate both autophagy and apoptosis. Pharmacological BH3 mimetics such as ABT-737, first discovered as inhibitors of antiapoptotic BCL-2s, caused regression of established tumors in mice [80]. Not surprisingly, this compound also affects the interaction between antiapoptotic proteins and Beclin 1. Recent studies show that ABT-737 competitively inhibits the binding of Beclin 1 to BCL-X_L_ and weakens the binding of Beclin 1 to BCL-2, thus freeing Beclin 1 to stimulate Beclin 1-dependent autophagy [81].

The interaction of Beclin 1 and BCL-2 is also regulated by c-Jun N-terminal protein kinase 1- (JNK1-) mediated BCL-2 phosphorylation and triggered by stress such as starvation [82]. Multisite (T69, S70, and S87) phosphorylation of a BCL-2 unstructured loop disrupts binding to BH3D-containing proteins such as Beclin 1 and BAX [83]. When cells are exposed to nutritional stress, phosphorylation initially disrupts the Beclin 1-BCL-2 interaction, upregulating autophagy to produce the missing nutrients and promote cell survival. However, prolonged starvation leads to increased levels of phosphorylated BCL-2, eventually disrupting the interaction with BAX, activating apoptosis, and leading to cell death [83]. 

### 4.2. Caspase-Mediated Beclin 1 Cleavage

The role of Beclin 1 in the interplay between apoptosis and autophagy can also be regulated by caspases. Caspase-mediated cleavage of Beclin 1 decreases cellular levels of Beclin 1 and consequently reduces levels of autophagy [50]. To date, three caspase cleavage sites have been identified in Beclin 1 : D133, D146, and D149 [49, 50, 84]. In one study [49], growth factor depletion initially upregulated autophagy inside Ba/F3 cells. However, sustained growth factor withdrawal reduced levels of autophagy and activated apoptosis. Further, after apoptosis was activated, Beclin 1 was found to be cleaved at D133 and D149. The resultant Beclin 1 fragment was incapable of mediating autophagy. Instead, the C-terminal fragments were found to localize to the mitochondria and sensitize Ba/F3 cells to growth factor deprivation-induced apoptosis. Interestingly, in a separate study [84] using HCT116 cells, Beclin 1 fragments generated by caspase-mediated cleavage at D133 and D146 during apoptosis did not induce either autophagy or apoptosis. Consistently, after chemotherapeutic treatment, in HCT116 cells expressing mutant D133A+D146A Beclin 1, the long-term survival rate was significantly improved compared to cells expressing wild-type Beclin 1. Further, xenograft tumors established using the mutant D133A+D146A Beclin 1 cells were also more resistant to chemotherapy. Thus, it is possible that preferential cleavage sites might be employed to generate different functional Beclin 1 fragments depending on cell types and treatments, which may have critical implications for cancer treatment. 

## 5. BIM and Its Role in Different Autophagy Stages

B-cell lymphoma 2-interacting mediator of cell death (BIM) is a potent proapoptotic protein. It may occur as three splice isoforms: BIM-short (BIM_S_), BIM-long (BIM_L_), and BIM-extra long (BIM_EL_). Different isoforms have different cellular functions. BIM_S_ and BIM_EL_ mainly function in apoptosis, while BIM_L_ has a more important role in autophagy [85]. BIM expression is upregulated by growth factor withdrawal, which mediates the inhibition of ERK1/2 and PKB signaling and the consequent dephosphorylation of the forkhead box O transcription factor, resulting in upregulation of BIM transcription [86, 87].

BIM triggers apoptosis by binding via its BH3 domain to BCL-2; preventing it from binding to and inhibiting BAX and BAK; consequently activating the intrinsic apoptotic pathway that leads to cell death [88]. Serum or growth factor triggered activation of ERK1/2 causes BIM_EL_ phosphorylation, releasing antiapoptotic BCL-2 homologs. Phosphorylated BIM_EL_ is recognized by E3 Ub ligase, resulting in BIM_EL_ ubiquitination and proteosomal destruction [89, 90]. As a result, cellular BIM_EL_ levels are reduced, apoptosis is inhibited, and cells can survive.

BIM, especially BIM_EL_, appears to inhibit autophagy independent of apoptosis activation, and this regulation occurs via diverse interactions of BIM with different proteins at multiple stages of autophagy. For instance, recently it was shown that BIM can directly interact with Beclin 1, and this interaction occurs at a site different from the BCL-2-binding region on Beclin 1 [91]. BIM-mediated regulation of autophagy is Beclin 1 dependent and can be disrupted by starvation. Additionally, previous studies [92] demonstrated that BIM_L_ is sequestered by dynein in healthy cells and dissociated upon an apoptotic stimulus. The interaction of BIM_L_ with dynein facilitates the loading and perhaps the fusion and positioning of lysosomes. Therefore, it is inferred that the absence of BIM leads to impairment of the later degradative phase of autophagy [85].

Thus, the activation of BIM can be used as a strategy for cancer therapy. One study has shown that AZD6244, which can repress the ERK1/2 signaling pathway, activates BIM expression, leading to cell death in colorectal cancer cells [93]. In another study, the mTOR inhibitor, rapamycin combined with MEK1/2 inhibitor, PD0325901, was found to increase BIM expression and promote cell death [94]. An evaluation of different isoform-specific effects of potential therapeutics targeting BIM will be important for their use in effective anticancer treatments.

## 6. UVRAG

UVRAG is a human homolog of yeast Vps38 [95]. Increased expression of UVRAG was shown to increase Beclin 1-PI3Kc3 interaction and PI3Kc3 lipid kinase enzymatic activity [96, 97]. Further, UVRAG was found to be essential for the localization of PI3Kc3 to the preautophagosomal structure and endosome [98]. Therefore, UVRAG was shown to be an important autophagy effector. UVRAG comprises an N-terminal polyproline disordered region, followed by a C2 domain, a CCD, and a large intrinsically disordered region [99]. The UVRAG CCD heterodimerizes with the Beclin 1 CCD, disrupting the Beclin 1 CCD homodimer and increasing autophagy levels in the cell. Binding of BCL-2 to Beclin 1 inhibits Beclin 1 binding to UVRAG, consequently inhibiting autophagy [100]. UVRAG appears to have a context-dependent role in cancer. It was shown to be mutated in microsatellite colon cancer cell lines and tumors, which consequently have reduced autophagy levels [101]. Conversely however, depletion of UVRAG in HEK cells did not affect autophagy but rather decreased epidermal growth factor receptor (EGFR) degradation, enhancing EGFR signaling and leading to tumorigenesis [102].

### 6.1. Interaction of UVRAG with BAX

Recently, UVRAG has been shown to function as an unusual BAX suppressor to regulate apoptosis [52]. The UVRAG C2 domain is responsible for binding BAX. UVRAG overexpression and increased interaction with BAX inhibits the exposure of the BAX N-terminus, and consequently, the mitochondrial translocation of BAX, mitochondrial membrane potential (MMP), and cytochrome c release, preventing apoptosis. Consistent with this effect, in human tumor cells such as HL60 and HCT116, suppression of UVRAG expression significantly increases apoptosis and decreases autophagy. Further, knockout of UVRAG in autophagy deficient *atg*5^−/−^ MEFs enhances doxorubicin-induced apoptosis. Therefore, it appears that UVRAG has a direct role in apoptosis regulation, which is independent of its role in autophagy. Thus, depending on the type and stage of cancer, therapeutics may target UVRAG to either increase autophagy levels within the cell or to inhibit its interaction with BAX and trigger apoptosis.

### 6.2. Interaction of UVRAG with Bif-1

Bif-1, a member of the endophilin B protein family, activates the conformational change of proapoptotic proteins BAX and BAK and subsequent cytochrome c release and caspase 3 activation during apoptosis [103]. More recently, it was shown that Bif-1 binds via its SH3 domain to the N-terminal polyproregion of UVRAG, while the Bif-1 BAR domain associates with membranes, facilitating autophagosome formation [96].

Bif-1 appears to function as a tumor suppressor, as knockout of Bif-1 leads to anchor-independent cell growth and tumorigenesis of HeLa cells through the activation of apoptosis [103]. Further, inhibition of autophagy caused by Bif-1 depletion has been shown to promote spontaneous lymphoma tumorigenesis in mice [96]. Therefore, novel cancer therapeutics may target UVRAG either to increase interaction with Beclin 1 and/or Bif-1, to increase autophagy, and facilitate degradation of oncogenic molecules; or to inhibit the UVRAG-BAX interaction, releasing BAX and triggering apoptosis. A combined approach, in which both autophagy and apoptosis are elevated to levels that facilitate cell death, may prove to be very powerful.

## 7. ATG12

As previously mentioned, the ubiquitin-like protein ATG12 is covalently conjugated to ATG5, and this conjugation is essential for autophagosome expansion. For many years, ATG5 was the only known target of ATG12, unlike most other known ubiquitin-like proteins modifiers.

### 7.1. ATG12–ATG3 Conjugation

In 2010, a novel target of ATG12 was identified: ATG3, the E2 enzyme involved in conjugation of phosphatidylethanolamine to ATG8, the other ubiquitin-like autophagy protein [53]. Surprisingly, disruption of ATG12-ATG3 conjugation did not affect starvation-induced autophagy but rather affected apoptosis regulation. Apoptosis induced by mitochondrial-uncoupling agents was reduced in cells lacking ATG12-ATG3 conjugation. This protection was found to correlate with increased expression of the antiapoptotic protein BCL-X_L_. Further, BCL-X_L_ inhibitors were able to induce a similar level of apoptosis in cells expressing either wild-type ATG3 or mutant ATG3 incapable of ATG12-ATG3 complex formation [53].

Factors that regulate whether ATG12 is conjugated to ATG5 or ATG3 would play a role in regulating the relative levels of autophagy and apoptosis. This may not only play a role in cancer development but also serve as a therapeutic target. Anticancer therapeutics may target the ATG12 conjugation process to selectively increase or decrease conjugation to either ATG5 or ATG3, thereby regulating relative autophagy or apoptosis levels. Less specific therapeutics that disrupt or increase all ATG12 conjugation, such as those that modulate ATG12 expression levels, could cause cancer cell death due to the combined effects of both pathways.

### 7.2. ATG12-Mcl-1 Interaction

Recently, ATG12 was shown to function as a positive mediator of apoptosis via interactions with the antiapoptotic BCL-2s [54]. ATG12 coimmunoprecipited with the antiapoptotic BCL-2 homolog, Mcl-1, and weakly with BCL-2. Further, the interaction between ATG12 and BCL-2s was disrupted by the coexpression of the proapoptotic, BH3-only protein BAD. Importantly, ABT-737, a BH3-mimetic inhibitor that specifically targets BCL-2/ BCL-X_L_, but not Mcl-1 [104, 105], disrupted the coimmunoprecipitation of ATG12 with BCL-2, but not with Mcl-1 [54]. Mammalian ATG12 homologs were found to contain a BH3 domain-like sequence motif within an intrinsically disordered region preceding the ubiquitin-like fold of ATG12. This motif appears to be an abnormal BH3D, because though it contains conserved residues important for binding to the hydrophobic surface groove on BCL-2 homologs [9], it also bears a proline which should prevent it from forming a regular *α*-helix like other BH3Ds. Consistent with a BCL-2 binding function, ATG12 mutations within the BH3-like motif did not affect ATG12-ATG5 conjugation, or the function of ATG12 in autophagy, but rather disrupted binding to BCL-2 homologs. In addition to the BH3-like motif, binding of Mcl-1 to ATG12 was found to require a second ATG12 site comprising an adjacent loop [54]. Notably, BCL-2 homologs were bound by free ATG12, but not the ATG12-ATG5 or the ATG12-ATG3 conjugates. Together, this information suggests that binding of ATG12 to the hydrophobic groove of antiapoptotic BCL-2s, especially Mcl-1, may prevent BCL-2s from binding to BH3 domain-containing proapoptotic proteins, thus triggering apoptosis.

Overexpression of Mcl-1 is observed in various cancers, rendering cells resistant to apoptosis induced by chemotherapy agents [106, 107]. Unlike other antiapoptotic proteins, Mcl-1 has a very short half-life, allowing an opportunity to combat these cancers by rapidly sensitizing Mcl-1-dependent cancer cells to chemotherapy-induced apoptosis upon inhibition of Mcl-1 [108]. Therapeutics that specifically target cancers involving Mcl-1 overexpression may function to improve binding of ATG12 to Mcl-1. Mcl-1 selectivity could be achieved by a detailed analysis of the binding of the abnormal ATG12 BH3D-like motif to Mcl-1 or by targeting the secondary ATG12 interaction site.

## 8. ATG5

As previously mentioned, the conjugation of ATG5 to ATG12 is essential for autophagy.

### 8.1. ATG5-FADD Interaction

Recently, a yeast two-hybrid screen showed that ATG5 also interacts with FADD [55]. This study also showed that certain stimuli, such as IFN-*γ*, upregulate ATG5 expression, resulting in autophagosome accumulation and cell death. In ATG5-overexpressing cells treated with IFN-*γ*, autophagosomes start to accumulate and then aggregate and fuse to form bigger vesicles. Eventually, most of the cells harboring aggregated vacuoles shrink and die. ATG5 mutants that cannot conjugate to ATG12 inhibit both IFN-*γ*-induced cell death and vacuole formation. However, ATG5 overexpression in FADD-deficient cells is insufficient for cell death, although vacuole formation is unaffected. Moreover, the autophagy inhibitor 3-methyadenine suppresses both ATG5-mediated vacuole formation and cell death, but the caspase inhibitor Z-VAD-fmk inhibits only cell death. Taken together, these findings indicate that in addition to its key function in autophagy, ATG5 may also have important roles in the regulation of apoptosis. Further, contrary to expectations, in this study, cell death appears to occur due to apoptosis rather than elevated autophagy and depends on the interaction of ATG5 and FADD. The ATG5-FADD interaction and its role in regulating this type of cell death provide additional targets for drug discovery and development of therapeutic strategies.

Altered ATG5 expression has been found in various types of cancers, including prostate cancers and gastrointestinal cancer [109, 110]. The complete lack or reduced expression of FADD in acute myeloid leukemia patients was shown to be associated with poor clinical outcomes [111]. The discovery of the ATG5-FADD interaction suggests that decreased expression of either ATG5 or FADD would impact both, autophagy and apoptosis, and therefore, these proteins likely play important and complex roles in cancer development. 

### 8.2. Calpain-Mediated ATG5 Cleavage

Calpains have been reported to cleave ATG5, and the cleaved ATG5 appears to provoke apoptotic cell death [56]. The cleavage product, an N-terminal ATG5 fragment with a relative molecular mass of 24 kD, is shown to translocate from cytosol to mitochondria. Both full-length ATG5 and truncated ATG5 are present in cells undergoing apoptosis. However, only the truncated ATG5 coimmunoprecipitates with the antiapoptotic protein BCL-X_L_, triggering cytochrome c release and caspase activation. Thus, truncated ATG5 loses its autophagy-inducing activity and instead appears to function as a proapoptotic protein that inhibits antiapoptotic BCL-2 homologs, resulting in the activation of mitochondria-dependent apoptosis.

Calpains catalyze the cleavage of numerous substrates, playing important roles in fundamental physiological processes, such as cytoskeletal remodeling and cellular signaling. Not surprisingly, calpain expression and its activity have been shown to be altered during the development of various cancers [112–114]. As calpain has now been shown to also mediate ATG5 cleavage, converting it from a proautophagic to a proapoptotic protein, it appears to also regulate the balance between autophagy and apoptosis. This adds a new and hitherto unexplored facet in the role of calpains in cancer development. For instance, cancer-triggering stimuli may suppress calpain expression, preventing generation of truncated ATG5, resulting in decreased apoptosis and the survival of cancer cells; conversely, stimuli that promote calpain-mediated ATG5 cleavage may be used to treat cancers. Therefore, the calpain-mediated ATG5 cleavage constitutes yet another checkpoint that may be targeted by cancer therapeutics.

## 9. p53: A Master Regulator of Autophagy and Apoptosis

The *TP53* gene encodes p53, a tumor suppressor [115] which is the most commonly mutated gene in human cancers, although some cancers retain wild-type p53. Stress-induced DNA or protein damage triggers repair mechanisms or programmed cell death, depending on the severity of the damage [116]. The response to DNA damage is regulated by p53, which plays a central role in cell cycle arrest and cell death. While p53 is an important transcription regulator, cytoplasmic p53 also has regulatory effects. 

**Figure 2:**
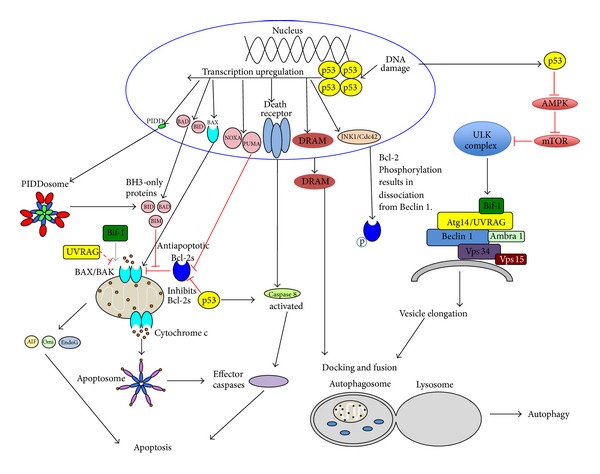
Regulation of autophagy and apoptosis by p53. Proteins and processes or interactions are represented as in Figure 1.

Both the extrinsic and intrinsic apoptotic pathways have been shown to be activated by p53 (Figure 2) [17]. In the extrinsic pathway, nuclear p53 increases the expression of death receptors such as the APO-1/Fas receptor [117] and the TRAIL receptor (DR4/5) [118]; while cytoplasmic p53 activates caspase 8 and caspase 3. In the intrinsic apoptotic pathway, nuclear p53 activates the expression of the proapoptotic proteins such as PIDD [17] and BH3-only proteins: PUMA, NOXA, BAX, and BID; leading to increased MMP, cytochrome c release, and activation of caspase-9 and caspase-8 [119]. Meanwhile, cytoplasmic p53 translocates to the mitochondria and forms a complex with BCL-2/BCL-X_L_ to liberate the proapoptotic proteins BAX and BAK [120]. p53 also activates the expression of APAF-1, a key component of the apoptosome [121]. 

In contrast to apoptosis upregulation by p53, cytoplasmic and nuclear p53 have contradictory roles in regulating autophagy (Figure 2). Cytoplasmic p53 inhibits autophagy through the activation of mTOR signaling via the inactivation of AMP kinase [122], while nuclear p53 activates autophagy by transcriptional activation of DRAM (damage-regulated autophagy modulator) which promotes the formation of autophagolysosomes [57]. In p53-induced apoptosis, the knock-down of DRAM leads to a decrease in cell death. In tumors with wild-type p53, DRAM mRNA is downregulated compared to tumors containing mutated p53, perhaps to mitigate the apoptosis-inducing function of p53 and facilitate survival of cancer cells [57]. In contrast to cytoplasmic p53, nuclear p53 activates kinases like Cdc42/JNK1, triggering BCL-2 phosphorylation at T56, S70, T74, and S87. Phosphorylated BCL-2 cannot bind to Beclin 1, allowing Beclin 1 to promote autophagy [123, 124]. Thus, nuclear p53 promotes autophagy.

The complicated role of p53 in regulating autophagy and apoptosis, makes it an important but complex target for cancer therapy. Cancer cells may be killed by therapeutics targeting p53 to increase apoptosis. For instance, in prostate cancer cells, resveratrol treatment activates MAPK, phosphorylating p53 at S15 and triggering p53-dependent apoptosis [125]. Overexpression of wild-type p53 induces both autophagy and apoptosis in SF126 cells, leading to cell death. This could be used as a strategy to treat cancer cells [126]. Conversely, p53 can facilitate cancer cell survival by modulating autophagy levels. For instance, in chronically starved HCT116 human colorectal cancer cells, p53 causes posttranscriptional downregulation of LC3. This allows basal levels of autophagic flux while preventing cell death associated with excessive autophagy, enabling cancer cell survival [127]. In contrast, the knockout of p53 leads to LC3 accumulation and culminates in apoptosis. So p53 increases cell fitness by maintaining autophagic homeostasis and modulating autophagy levels according to environmental changes [126, 127]. The p53/HMGB1 complex also crossregulates autophagy and apoptosis in human colorectal cancer cells [128]. The knockout of p53 increases cytoplasmic HMGB1 levels, facilitating cell survival through autophagy activation. Conversely, loss of HMGB1 increases cytoplasmic p53 levels and p53-induced apoptosis.

Given the complex role of p53 in regulation of autophagy and apoptosis, as well as the varied effects of different truncated and mutant forms of p53 on these pathways, it is not surprising that p53 plays a complicated role in cancer. Only wild-type p53 has been conclusively shown to trigger apoptosis; therefore, it is particularly important to consider that therapeutics targeted against the wild-type protein may be ineffective in cancer cells which contain mutant p53. For instance, one study has shown that in estrogen-positive breast cancer cells, expression of a truncated p53 lacking the C-terminal 102 amino acids increases BCL-2 expression by alleviating the repression by endogenous wild-type p53, thus decreasing apoptosis [129].

## 10. The Regulation of Autophagy by Antiapoptotic Viral BCL-2 Homologs

Some virus-encoded proteins also modulate the crosstalk between autophagy and apoptosis in order to facilitate virus survival and amplification. Homologs of the antiapoptotic cellular BCL-2s are encoded by all *γ*-herpesviruses (*γ*HV) [130, 131], as well as some other viruses like the African swine fever virus and some pox viruses [132–134]. Despite low sequence conservation between the various cellular and viral BCL-2 homologs, all those with known 3D structures share the same fold, indicating that they are homologs. Viral BCL-2s are thought to sustain host cell viability by preventing cell death, in order to maximize viral replication [135, 136]. Further, viral BCL-2s contribute to establishment of latency, the emergence from latency, and the establishment of chronic, persistent infections [137, 138].

*γ*HVs, including important human pathogens such as Epstein Barr virus (EBV), Kaposi's sarcoma-associated HV (KSHV), and the murine *γ*HV68, are associated with lymphoproliferation and cancer. EBV is implicated in the pathogenesis of a number of human malignancies of epithelial and lymphoid origin and a number of lymphoproliferative diseases in immunocompromised hosts [139, 140], while KSHV is involved in the etiology of Kaposi sarcoma tumors [141]. EBV encodes two BCL-2 homologs: BHRF1 and BALF1. BHRF1 binds to BH3 domain-containing proapoptotic proteins, including BIM, BID, PUMA, and BAK. BHRF1 expression renders a mouse model of Burkitt lymphoma untreatable [142]. Unexpectedly, the other BCL-2 encoded by EBV, BALF1, fails to protect cells against apoptosis. Instead, it appears to inhibit the antiapoptotic activity of BHRF1 [139]. KSHV-encoded BCL-2 was also shown to bind to the BH3Ds of BAX and BAK with affinities significantly lower than cellular BCL-2s [143]. However, a separate study found that KSHV BCL-2 failed to heterodimerize with cellular BAX and BAK proteins, although its overexpression leads to the inhibition of Sindbis Virus-induced apoptosis [144]. Murine *γ*HV68 also encodes an antiapoptotic BCL-2 homolog, M11, that inhibits apoptosis induced by anti-Fas antibody and by TNF-*α* [136]. Like the cellular BCL-2s, viral BCL-2s also bear a hydrophobic surface groove that is responsible for binding to the BH3D of various proapoptotic proteins [61, 62, 142, 143].

Therefore, it is not surprising that *γ*HV BCL-2s have now been shown to bind to the Beclin 1 BH3D to function as potent autophagy inhibitors. KSHV BCL-2 was shown to block Beclin 1-dependent autophagy in both yeast and mammalian cells [76]. Subsequently, *γ*HV68 M11 was shown to also bind to Beclin 1 and inhibit autophagy. Structures of M11 in complex with the Beclin 1 BH3D show that the Beclin 1 BH3D binds to the hydrophobic surface groove on M11 [61, 62], similar to the mode by which various BH3Ds had been shown to bind to cellular BCL-2s. M11 also binds to most proapoptotic proteins except BAD, BIK, and BAK [145]. Thus, M11 inhibits both apoptosis and autophagy by binding to BH3Ds from proapoptotic proteins and the proautophagic protein, Beclin 1 [62]. However, unlike the cellular BCL-2s, the inhibitory activity of *γ*HV BCL-2s does not appear to be downregulated by cellular phosphorylation, allowing them to constitutively inhibit autophagy and apoptosis [82].

As the *γ*HV BCL-2s are essential for oncogenicity of these viruses, they are important targets for therapeutics targeting these viruses. Similar to the cellular BCL-2s, peptidomimetic molecules that selectively target the BH3D-binding grooves of the *γ*HV BCL-2s would serve to increase autophagy, enabling xenophagic degradation of the virus, as well as to increase apoptosis, enabling apoptotic destruction of the virus along with the host cell. Further, molecules that selectively target the *γ*HV BCL-2, but not the cellular BCL-2s, especially molecules that selectively disrupt the interaction with Beclin 1, may allow selective clearance of the virus, without destroying the host cell. 

## 11. Summary

Autophagy and apoptosis both function as anticancer pathways. Defective apoptosis leads to reduced cell death, and consequently, this is a common feature in the development and progression of cancer. Initially, autophagy was also identified as a tumor suppressor pathway, as in normal cells it facilitates the degradation of oncogenic molecules. However, since then, autophagy has been shown to have a much more complicated role in cancer. Autophagy was assigned additional roles in tumor suppression due to the involvement of autophagy proteins in type II cell death. Indeed, type II cell death has been shown to contribute to many cancer treatments [146, 147]. However, the discovery of several nodes of molecular crosstalk between autophagy and apoptosis, combined with the requirement for apoptosis proteins even for type II cell death, appears to indicate that type II cell death may result merely from an upregulation of apoptosis by selected autophagy proteins [148]. Adding further to this confusion, in nutrient deprivation or therapeutic stress conditions, autophagy may actually support the survival of cancer cells. Thus, unlike apoptosis, the role of autophagy in cancer appears to be very diverse, dictated primarily by cellular contexts. In addition to the cross-regulation mechanisms reviewed here, it is likely that future research may identify still more mechanisms of crosstalk between autophagy and apoptosis, as well as between autophagy and other pathways important in cancer. These multiple molecular nodes of crosstalk present many opportunities for selective therapeutic intervention in different cancers. Thus, research investigating these mechanisms of cross-regulation that enables a complete understanding of the coordinate regulation of autophagy and apoptosis is essential for the rational design of successful anticancer therapeutics.
